# Mucosal HSV-2 Specific CD8+ T-Cells Represent Containment of Prior Viral Shedding Rather than a Correlate of Future Protection

**DOI:** 10.3389/fimmu.2013.00209

**Published:** 2013-07-29

**Authors:** Joshua T. Schiffer

**Affiliations:** ^1^Department of Medicine, University of Washington, Seattle, WA, USA; ^2^Vaccine and Infectious Diseases Division, Fred Hutchinson Cancer Research Center, Seattle, WA, USA; ^3^Clinical Research Division, Fred Hutchinson Cancer Research Center, Seattle, WA, USA

**Keywords:** HSV-2, mathematical modeling, CD8+ T-cells, mucosal immunology, tissue resident memory cells

## Abstract

It is largely unknown why certain infected hosts shed Herpes Simplex Virus-2 (HSV-2) more frequently and have more severe disease manifestations than others. One idea is that different density or functional capacity of tissue resident effector memory CD8+ T-cells between infected persons may explain phenotypic variability. To generate hypotheses for contrasting shedding patterns in different infected hosts, a spatial mathematical model was employed to evaluate the effects of variability in tissue resident effector memory CD8+ T-cell response, and HSV-2 replication and spread, on viral shedding rate. Model simulations suggest that high levels of CD8+ T-cells in the mucosa do not necessarily indicate a protective phenotype but rather an effective response to recent shedding. Moreover, higher CD8+ T-cell expansion rate and lower viral replication rate, which correlate with better short-term control, may have only a minor impact on long-term shedding rates. Breakthrough shedding occurs under all sets of model parameter assumptions, because CD8+ T-cell levels only surpass a protective threshold in a minority of genital tract mucosal micro-regions. If CD8+ T-cell levels are artificially increased using an immunotherapeutic approach, better control of shedding is predicted to occur for at least a year. These results highlight the complex co-dependent relationship between HSV-2 and tissue resident CD8+ lymphocytes during the course of natural infection.

## Introduction

A question at the core of infectious diseases research is why different patients exhibit heterogeneous disease severity when exposed to equivalent pathogens. This question is especially relevant for Herpes Simplex Virus-2 (HSV-2) infection, which is characterized by variable genital tract shedding, as well as recurrent genital lesions in many, but not all patients. Approximately 20% of patients are completely asymptomatic despite shedding virus (1, 2); on the other extreme, some patients recur more than 10 times per year, and others suffer from recurrent meningitis (3). Several informative cross-sectional studies have identified specific innate immune deficits that correlate with severe disease outcomes during HSV-1 infection (4, 5), which causes both oral and genital ulcers, and has close genetic homology to HSV-2. Other studies demonstrated that at a broad level, deficits in T-cell functionality correlate with higher recurrence rates (6, 7). Yet, these studies do not allow a full mechanistic explanation for disease variability, and ignore important non-linear dynamic features of the immune response as well as viral replication. It is not understood, even at a basic level, why some patient shed more than others, and why many patients suffer while others remain completely asymptomatic.

There is currently an intense focus on the role of tissue resident CD8+ effector memory T-cells in rapidly containing HSV-2 infection (8–11). In humans, these cells are present in high local abundance at the site of viral replication during genital ulcers (12, 13). Importantly, tissue resident CD8+ lymphocytes persist for months at the site of HSV-2 release from neurons into the genital tract and at the single cell level, retain an activated effector memory phenotype, even in the absence of viral reactivation (14, 15). In animal models, tissue resident CD8+ T-cells can be “pulled” from blood to tissue using local inflammatory mediators, a promising observation for vaccine developers (16). Upon arrival to the mucosa, these cells actively patrol between epithelial cells (17), and rapidly contain HSV replication with re-challenge of virus (9, 18).

It is therefore tempting to invoke variability in function and number of CD8+ T-cells as a key determinant of inter-subject shedding variability (19). Dense imprints of HSV-2 specific CD8+ lymphocytes in genital tissues may represent a protective phenotype against subsequent high levels of genital shedding. Yet, the temporospatial dynamics of tissue resident cells toward HSV-2 are complex because HSV-2 reactivation occurs approximately every week, implying more leaky control of HSV over brief time scales (20): a high abundance of tissue resident HSV-2 specific CD8+ T-cells may simply reflect recent local containment of virally infected cells rather than prospective immunologic protection. The relative stability of shedding rates in humans over decades of infection supports this idea (21).

Studying this problem in humans is complicated by the fact that immunologic sampling of the genital tract is limited to millimeter tissue sections, and CD8+ T-cells expand and contract within hundreds of genital tract microenvironments, resulting in spatially heterogeneous potential for viral growth (22). While some genital tract areas may be protected, other potential regions of viral reactivation lack protective T-cell immunosurveillance. This spatial variability is an extremely important, but typically overlooked, feature of the mucosal immune response. While HSV-2 severity can be compared between study subjects using total genital tract shedding rate and lesion rate, as outcome measures (23), the overall intensity and spatial variability of the immune response is not easily measured at the whole tissue level.

In this study I use a published mathematical model to simulate heterogeneous shedding rate in different infected persons while also developing predictions regarding the relationship between viral shedding and CD8+ T-cell spatial density and functionality over long time frames. The model describes competition between HSV-2 replication in mucosal epithelial cells, and CD8+ T-cell elimination of these infected cells (19, 23, 24). Because HSV-2 lesions consists of multiple ulcers, viral replication is assumed to be widespread across the genital tract (25), and CD8+ T-cell expansion is assumed to localize to micro-regions of viral replication (12, 13). Equations are structured to allow multiple, spatially discrete, concurrent foci of replication and immunological containment. Model parameters characterize rates of viral replication and spread, death rate of infected cells, and kinetics of CD8+ T-cell expansion, decay, and cell lysis. Several spatial phenomena are captured by the model including widely dispersed viral release from neurons into multiple regions of the genital tract, seeding of adjacent regions of genital skin by virus from a single ulcer, and measurement of immunologic distance between newly seeded ulcers (22). The model’s key emergent property is shedding rate, which is influenced to varying degrees by all of these biologic processes.

The model previously reproduced detailed kinetic features from merged data consisting of 14,685 genital swabs and 1,020 shedding episodes from 531 study participants (22). It therefore provides a general biologic framework to explain general shedding episode patterns that are evident in most infected persons. However, because episode initiation is difficult to predict, episode severity varies dramatically over 30 to 60-day sampling periods in clinical studies, and viral load trajectories are highly erratic and non-linear, the model is not easily fit to data from individual patients, and has not been used as a tool to explain inter-subject shedding variability.

Here I perform global sensitivity analysis to isolate model parameters that have the greatest impact on HSV-2 genital shedding rate. In simulations, high levels of overall CD8+ T-cell density do not correlate with better control of virus. In fact, high shedding appears to drive the frequency of mucosal T-cell turnover. Regardless of shedding rate, large sections of the genital tract have low enough CD8+ T-cell levels to remain susceptible to more severe episodes, highlighting that HSV-2 capitalizes on spatial heterogeneity of local immunity to allow occasional high-titer, prolonged shedding episodes. As in clinical studies, an important simulated determinant of long-term shedding rate is episode rate, which in turn is strongly influenced by rate of release of HSV-2 from neurons into the genital tract. Surprisingly, parameter values, such as increased mucosal CD8+ T-cell expansion rate or decreased viral replication rate in keratinocytes, that imply more robust short-term virologic control in genital mucosa, have little impact on long-term shedding rate. These results suggest that while tissue resident CD8+ T-cells are critical in HSV-2 containment during the natural history of infection, high local tissue density does not necessarily imply better control. Yet, if natural dynamics of infection are perturbed with an immunotherapy leading to an increase in overall density of tissue resident CD8+ T-cells, then shedding rate is predicted to decrease for at least a year.

## Materials and Methods

### Analysis of shedding data

The data informing model design was from studies in which HSV-2 seropositive participants performed swabs of the genital tract every 24 h, whether lesions were present or not, for a minimum of 30 days. Swabs were processed for quantitative HSV DNA polymerase chain reaction (PCR) using PCR. Detection of HSV DNA was performed using a well-validated collection and detection method (26–28) and was expressed as copies per milliliters of medium.

**Figure 1:**
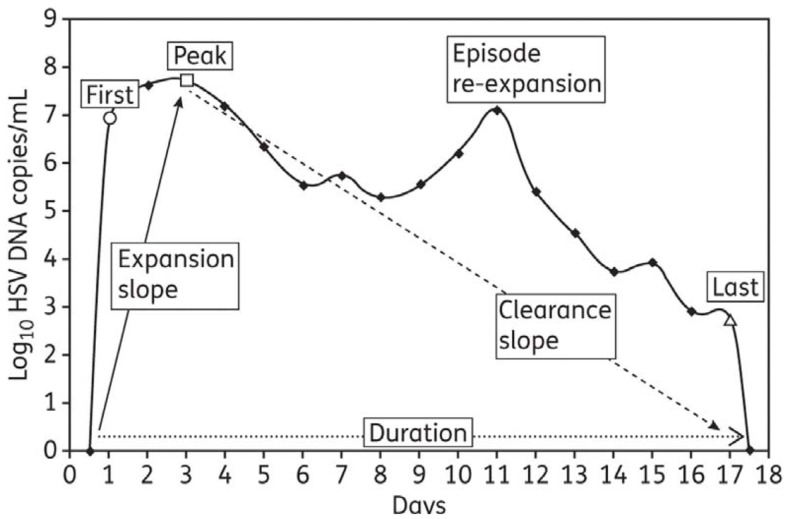
**Shedding episode classification scheme: from Schiffer et al., J Antimicrob Chemother. 2011;66(11):2593–600. (29)**.

To provide a detailed quantitative summary of episodic shedding, eight summary measures were used: (1) quantitative shedding rate was evaluated on a per swab basis with a frequency histogram that stratified viral load into bins according to increases in log-value (10^2^–10^3^, 10^3^–10^4^… HSV DNA copies); seven measures applied to specific episode characteristics (Figure 1): four frequency histograms were used to describe heterogeneity in (2) peak, (3) first, and (4) last positive HSV DNA copy number within an episode (bins were again separated by log-value of HSV DNA), as well as (5) episode duration (bins separated by number of days); median episode measures included slopes from (6) initiation to episode peak and (7) peak to termination, as well as (8) episode frequency. Initiation to peak slopes were calculated with the assumption that episodes initiated 12 h before the first positive swab respectively, and peak to termination slopes with the assumption that episodes ended 12 h after the last positive swab respectively (22, 29).

### Model simulations

**Figure 2:**
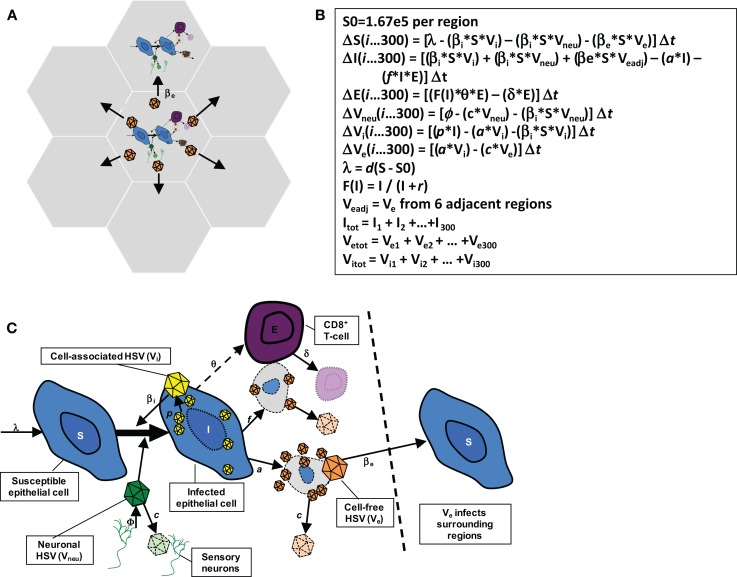
**Spatial mathematical model**. **(A)** Seven of 300 micro-regions are shown to indicate that cell-free virus from a single region can infect adjacent regions. **(B)** Model equations. **(C)** Model diagram indicating viral production, spread, and local CD8+ T-cell response: from Schiffer et al., eLife 2013;2:e00288. (22).

The model in this paper has been described in detail elsewhere (22) and is diagramed in Figure 2. The model consists of 300 possible micro-regions of infection, linked by (1) the ability of neurons to randomly release virus into any of the 300 regions, (2) the ability of virus from a herpetic ulcer in one region to seed a new herpetic ulcer in an adjacent region, and (3) the ability of CD8+ T-cells from one region to influence viral containment in an adjacent infected region. The 300 micro-regions are arrayed in a two-dimensional matrix of hexagons such that cell-free particles from a region can only infect a maximum of six other regions (Figure 2A), thus limiting rate of HSV-2 spread from region to region within the genital tract. For edge regions, there are only four contiguous regions at risk. These limitations on creation of new ulcer spread are imposed to account for the clustered nature of genital ulcers in infected people.

The HSV-2 replication cycle and immunologic response to HSV-2 infected cells within a single genital tract microenvironment can be referenced to each of the model’s parameters (Figures 2B,C). During HSV-2 *reactivation*, virions are released from neuronal endings at the dermal-epidermal junction at a certain rate (ϕ). Released HSV-2 survives in the genital tract for a defined duration (1/*c*) during which time it can transfer from neurons and infect local epithelial cells. Viral infectivity [1/(β_i_ × *S*)] is roughly defined as the number of viruses that are needed to infect one epithelial cell per day assuming the constant presence of viruses and a full complement of susceptible cells. For all of the models in this manuscript, there is some degree of target cell limitation within each micro-region, by virtue of susceptible cells decreasing relative to total number of cells with time.

I assumed that if an epithelial cell becomes infected, then it will die via direct lysis if it survives long enough to become packed with viruses (lifespan = 1/*a*), or via CD8+ lymphocyte (*E*) mediated killing [lifespan = 1/(*f* × *E*)]. Lymphocyte killing efficiency (*f* ) is defined as the number of infected cells cleared by one CD8+ lymphocyte cell per day *in vivo*. If an epithelial cell evades CD8+ lymphocyte mediated killing for the entire duration of cellular infection, it will produce a total of *p/a* viruses: *p* is the rate of viruses produced by an infected cell per day. Re-growth of susceptible keratinocytes occurs according to a growth rate, λ or *d* × (*S*_0_ − *S*) with growth limited by *S*_0_, the carrying capacity of the system.

The formation of a genital lesion is accompanied by rapid accumulation of localized CD8+ lymphocytes at the dermal-epidermal junction at a peak rate (θ), followed by slow decay of these cells over a period of months after lesion healing (lifespan = 1/δ). The CD8+ replication rate in our model is saturated at θ, and θ/2 occurs when infected cells are equal in number to parameter *r*, which represents how many epithelial cells need to be infected prior to half-maximal CD8+ expansion. Therefore, parameter *r* defines how rapidly immune cells recognize and respond to viral antigens on the surface of infected cells. Local replication of CD8+ T-cells rather than trafficking from other sites is the most mathematically likely means of viral control (19, 24). For each fitting simulation, there is included a minimum value of *E* within each region to reflect low numbers of CD8+ T-cells observed in nearly all biopsy studies to date.

Cell-associated HSV-2 is differentiated from cell-free HSV in the model and it is assumed that cell-associated particles (*V*_i_) can passage from cell-to-cell within one microenvironment as soon as they form within a cell (30). This process allows viral ulcers to spread in a radial fashion. Cell-associated particles (*V*_i_) convert to cell-free virus (*V*_e_) after infected cells rupture (Figure 2C). Because swabs probably do not remove most cell-associated particles and infected cells (otherwise swabbing would mitigate shedding), *V*_e_ is fit to the data. Cell-free particles can initiate formation of new plaques within adjacent regions by local seeding in our model, and are assigned an infectivity parameter (β_e_). Parameter (ε) is included to account for delay in viral production within secondary plaques after seeding.

The 300 regions are linked immunologically according to parameter ρ. When an ulcer is initiated in a region (infection of a single epidermal cell), the CD8+ T-cell density is assumed to include a certain proportion of CD8+ T-cell density in surrounding regions: we include this parameter to acknowledge that there are in fact no discrete immunologic borders between regions as assumed in the model. If ρ = 0, then CD8+ T-cell density in a region is independent of surrounding regions. This implies that all immunity is localized only to an area with viral replication. If ρ = 1, CD8+ T-cell density in a region is assumed to equal the average from surrounding regions.

The reproductive number [*R* = (*p* × β_i_ × *S*_0_)/(*a* + *fE*) × *c*] is continually updated during each simulation. The reproductive number is the average number of cells that an infected cell would infect assuming the presence of CD8+ T-cells. For the spatial model, which divides the genital tract into 300 separate regions that are susceptible to viral replication, I calculated *R* separately within each region.

A feature of the model is sensitivity to CD8+ T-cell density at episode onset, which has an important effect on amount of virus produced per episode (19). To avoid creating a bias on model outcomes due to initial CD8+ T-cell values, the model was simulated with its particular parameter set for 365 days, and then the CD8+ T-cell values in each region were used as the starting values for the recorded simulation. Each simulation started with zero infected cells and viruses.

### Sensitivity analysis

Two hundred unique parameter sets were generated by randomly selecting each parameter value using Latin hypercube sampling from probability distribution functions of each parameter (pdfs). The pdfs were constructed by normalizing around best-fit values from model fitting in previous simulations of the model (22, 24). The 10 parameters are included in Table 1. Viral replication rate was converted to a log-value prior to normalization to expand variability of this parameter. Simulations were performed over 10 years and parameter values were correlated with several outcomes including shedding rate, episode rate, and area under the curve which was calculated by measuring the sum of [(*V*_e,t_ + *V*_e,t + 0.001_)/2] × 0.001 over the course of the simulation.

**Table 1:** **Parameter ranges used for sensitivity analysis**.

Parameter	Units	Symbol	Mean	SD
Cell-associated HSV infectivity	DNA copy days/cell (viruses needed per day to infect one adjacent cell)	β_i_	6.3e − 8	7.5e − 9
Cell-free HSV infectivity	DNA copy days/cell (viruses per day to initiate one ulcer)	β_e_	2.2e − 11	2.5e − 12
Epidermal HSV replication rate	log 10 HSV DNA copies/cell/day	*p*	5.05	0.05
Neuronal release rate	HSV DNA copies/day/genital tract	ϕ	68	11
Free-viral decay rate	Days^-1^ (half-life, hours)	*c*	8.4	0.7
Maximal CD8+ T-cell expansion rate	Days^-1^	Θ	2.6	0.35
CD8+ T-cell decay rate	Days^-1^ (half-life, days)	δ	1.4e − 3	1.4e − 4
CD8+ T-cell local recognition	Infected cells at which Θ is half-maximal	*r*	39	4
CD8+ regional co-dependence	0 = no co-dependence, 1 = full co-dependence	ρ	0.72	0.07
Viral production lag	Days	ε	0.8	0.15

*Normal distributions were assumed*.

To allow for more parameter variability, I expanded the standard deviation of each parameter twofold and conducted a second otherwise equivalent set of global sensitivity analyses. These results did not alter the trends observed with a narrower standard deviation and as such are not included in the manuscript.

### Assessing mathematical model fit to empirical data

All simulations were performed using C++. Model simulations with each of the unique parameter sets were assessed for their fit to a cumulative measure of the eight summary measures (42 data bins). The model was solved stochastically due to the random nature of shedding episode initiation and clearance, and to account for frequent presence of low numbers of infected cells: at each time step, integer values for equation terms were drawn randomly from binomial distributions. To assess degree of fit between model numerical output and empirical data required prolonged simulations of 10-year duration to minimize fluctuations in output due to stochastic effect. Model variables were updated at a narrow time interval (0.001 days). However, for assessing fit to data, we assembled the modeled data exactly as it was gathered in the clinical protocols by sampling every 24 h.

Each unique parameter set was assigned a *least squares fit score* by the following methods. First, I assigned each of the summary measures [(1) episode rate, (2) episode duration, (3) median initiation to peak slope, (4) median peak to termination slope, (5) first positive copy number of episodes, (6) last positive copy number of episodes, (7) peak positive copy number of episodes, and (8) per swab quantitative shedding] a weighting factor to ensure that each summary measure carried an equivalent weight. Using the empirical data, the mean value of bins within each of the five histograms [(1) episode duration, (2) first positive copy number of episodes, (3) last positive copy number of episodes, (4) peak positive copy number of episodes, and (5) quantitative shedding] was calculated; the inverse square of this value was then used to generate an *initial weighting factor*, which was then divided by the number of bins within the histogram such that each bin was assigned a *bin weighting factor*. The three median measures [(1) episode rate, (2) median initiation to peak slope, and (3) median peak to termination slope] only contained one bin such that the *initial weighting factors* were equal to the *bin weighting factors*.

For each bin, the difference between the empirical data and model output was squared and multiplied by the *bin weighting factor* for the bin, to arrive at a *bin score*. Each simulation with a unique parameter set was given a *least squares fit score* equal to the sum of these 42 *bin scores* with a lower score representing better model fit. Unique parameter set simulations with the lowest *least squares fit score* tended to capture all critical dynamical features of HSV-2 shedding.

## Results

### Shedding rate is the most important clinical outcome

**Figure 3:**
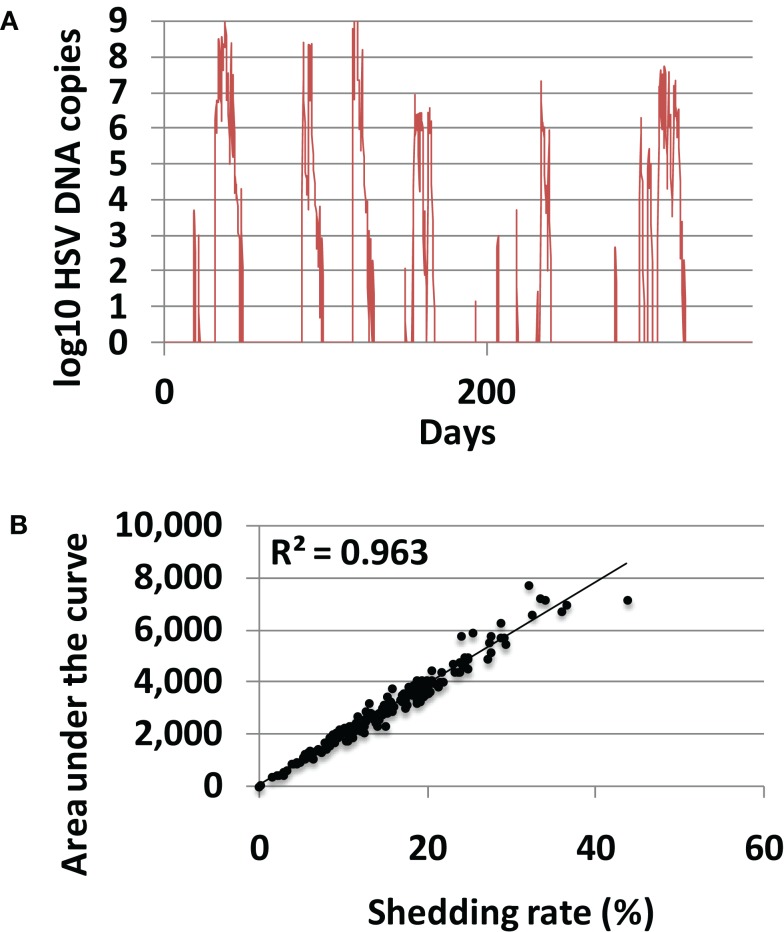
**Shedding rate is the most relevant measure of HSV-2 severity**. **(A)** Typical model simulation demonstrating 365 days of shedding. **(B)** Results from 200, 10-year model simulations, each with randomly selected parameter values. Shedding rate correlates tightly with a cumulative episode area under the curve indicating that shedding rate also highly predicts quantitative levels of viral shedding.

In 200 model simulations, each with a unique randomly selected parameter sets, shedding was episodic and episodes were heterogeneous according to duration and viral production (Figure 3A). Shedding rates were highly variable, ranging from 0 to 44% akin to rates described in clinical studies (26–28). Shedding rate correlated precisely with viral area under the curve for each simulation (Figure 3B), confirming a finding from empirical datasets that shedding rate strongly predicts a composite measure of frequency and quantity of shedding. Shedding rate is therefore the most convenient and reproducible measure of disease severity and transmission risk. Shedding rate is also a fundamental emergent property of the model and therefore serves as the key outcome for the remainder of this study.

### Model parameters are optimized for high shedding at the population level

**Figure 4:**
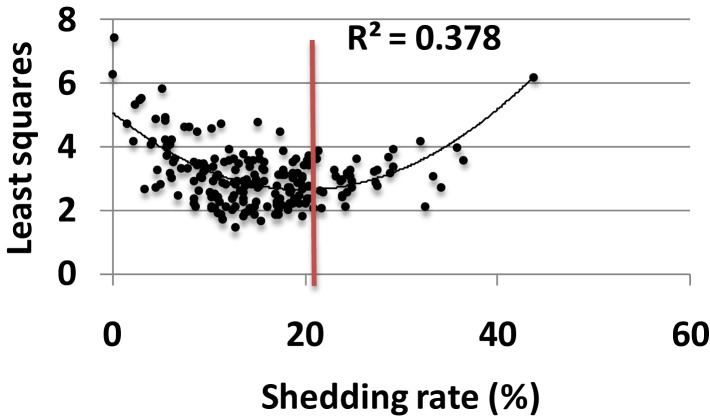
**Model parameters are optimized for high shedding**. Results from 200, 10-year model simulations, each with randomly selected parameter values. Simulations with best fit to all features of shedding episode features (rate, duration, early expansion and late decay kinetics, peak viral production and, expansion and decay slopes) lead to lowest least squares values (*y*-axis) and also most closely approximate the population levels shedding rate of 18% (red line); only 40 of 200 parameter sets lead to shedding rate>18%, implying that viral and immunologic parameters are relatively well calibrated to allow maximal shedding. Black line is a Lowess smoothed curve.

Each parameter set was assessed for its fit to a large empirical dataset of 14,685 swabs from 531 study participants (22). The eight characteristics of shedding (see Materials and Methods) were measured in each simulation and compared to the empirical data. Parameter sets with closets fit to the data (by virtue of lowest least squares fitting test) also were closest to the actual shedding rate (Figure 4). As simulated shedding rate decreased below 18%, model fit generally decreased accordingly. Most parameter sets resulted in shedding rates below that of the population level value of 18%, suggesting that viral replication and immune response parameters are relatively optimized to maximize shedding (Figure 4).

### High tissue CD8+ T-cell levels do not predict lower shedding rates

**Figure 5:**
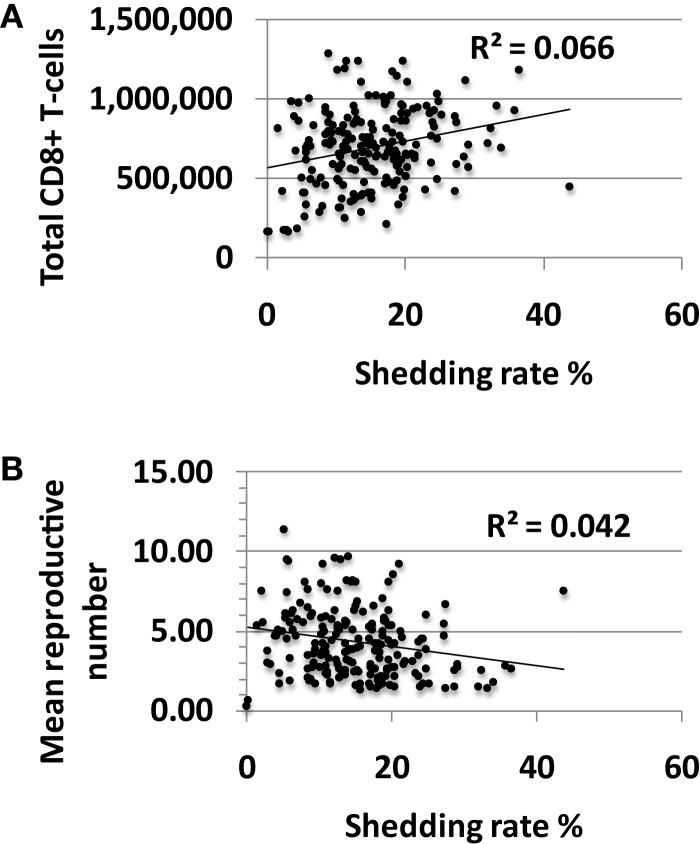
**High shedding rate partially drives higher levels of CD8+ T-cell inflammation**. Results from 200, 10-year model simulations, each with randomly selected parameter values. **(A)** Shedding rate has a positive, rather than a negative correlation with the average total amount of CD8+ T-cells in the genital tract over a 10-year period. **(B)** The average growth potential (reproductive number) of all 300-model regions over 10 years in high shedders, indicating that during the natural history of disease, high shedding rate actually correlates with a slightly lower average growth potential for virus across time and space.

There was limited association between shedding rate and CD8+ T-cell density averaged over 10 years with a small positive correlation between these two outcomes (Figure 5A). The reproductive number in a single model region derives from the CD8+ T-cell density with high levels of CD8+ lymphocytes driving the reproductive number less than one. Under this condition, immediate containment of infected cells is favored while much more extensive local spread of HSV-2 to thousands of infected cells occurs at higher reproductive numbers. The reproductive number, or potential for viral growth, averaged across the 300-model regions over time, correlated inversely with shedding rate (Figure 5B) but was significantly greater than one for all simulations with shedding indicating high general potential for viral growth in all shedders (Figure 5B).

### High shedding rate predicts a more dynamic CD8+ T-cell state

**Figure 6:**
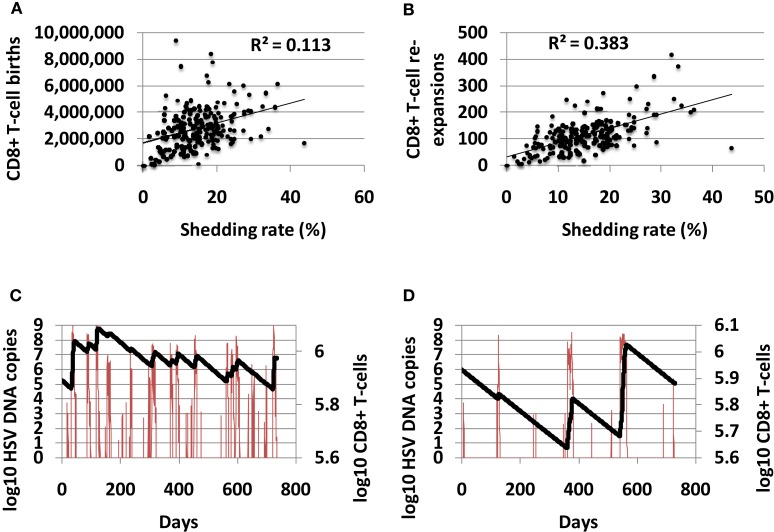
**High shedding rate predicts a highly dynamic immunologic state with more frequent CD8+ T-cell turnover**. **(A)** Shedding rate predicts number of CD8+ T-cell births over 10 years, and strongly predicts **(B)** number of CD8+ T-cell expansion events. **(C)** An example of a high shedder (HSV DNA in red and CD8+ T-cells in black) with frequent CD8+ T-cell reconstitution events. **(D)** An example of a low shedder (HSV DNA in red and CD8+ T-cells in black) with less common CD8+ T-cell reconstitution events.

The total number of CD8+ T-cell births over 10-year correlated positively with shedding rate, indicating that CD8+ births are responsive to high level shedding (Figure 6A). The number of CD8+ T-cell expansion events (defined as an increase of at least 10 CD8+ T-cells) also correlated with shedding rate (Figure 6B). A higher number of shedding episodes increased the frequency of CD8+ lymphocyte expansion (Figures 6C,D) leading to a more dynamic inflammatory state.

### Spatial heterogeneity of CD8+ T-cell density and immunologic readiness is present in all infected persons and does not impact shedding rate

**Figure 7:**
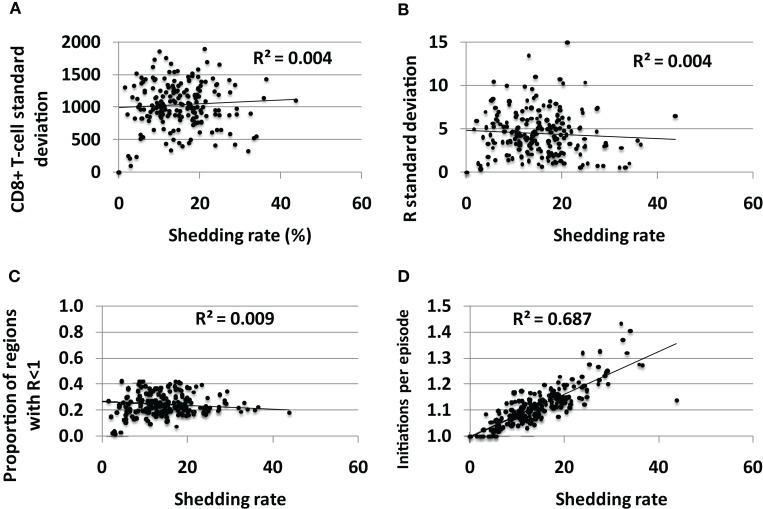
**Spatial heterogeneity in CD8+ T-cell levels across the genital tract does not explain variable shedding rates between infected persons**. The average **(A)** CD8+ T-cell variability between regions and **(B)** reproductive number variability between regions over time, does not differ between low and high shedders. **(C)** Genital tract regions with potential for high shedding (*R* < 1) predominate among low and high shedders. **(D)** High shedders have episodes with more region initiations per detected episode than low shedders: therefore, initiation of shedding in multiple regions per episode does not appear to decrease shedding rate.

The average standard deviation of CD8+ T–T-cell density (Figure 7A) and the reproductive number (Figure 7B) across the 300 spatially discrete model regions did not vary according to shedding rate, though the variability between regions was generally high for all shedders suggesting substantial spatial heterogeneity of immune readiness in persons with chronic HSV-2 infection. Fewer than 50% of genital tract regions had reproductive number<1 for all sets of parameters (Figure 7C), suggesting that most of the genital tract can support high levels of HSV shedding at a single point in time. This is consistent with genital biopsy studies that reveal low CD8+ T-cell density in non-shedding regions (12, 13).

If the immunologic response to HSV-2 protected broad regions of at risk tissue, then low CD8+ T-cell density regions in the genital tract could be viewed as a limited ecologic “resource” for the virus. To maximize shedding would require that HSV not over utilize this resource by spreading rapidly to all susceptible regions, thereby inducing high CD8+ T-cell levels across the genital tract. Theoretically, if episode initiation rate is too high due to frequent release of HSV from neurons, then many spatially separate episodes in the mucosa might overlap. Some shedding might be wasted, as simultaneous shedding in different sites does not increase the shedding rate. We measured number of average episode initiations per episode to assess this phenomenon. Higher initiations per episode correlated tightly with shedding rate in simulations, indicating that overlapping initiations, while common, do not occur frequently enough to meaningfully lower shedding rate (Figure 7D). Therefore, low CD8+ T-cell regions do not appear to be a limited resource for HSV-2 under any parameter conditions, and multiple regions with low CD8+ T-cell density allow breakthrough episodes in low and high shedders alike.

### Limited effect of mucosal CD8+ T-cell functional parameters on long-term shedding rate

**Figure 8:**
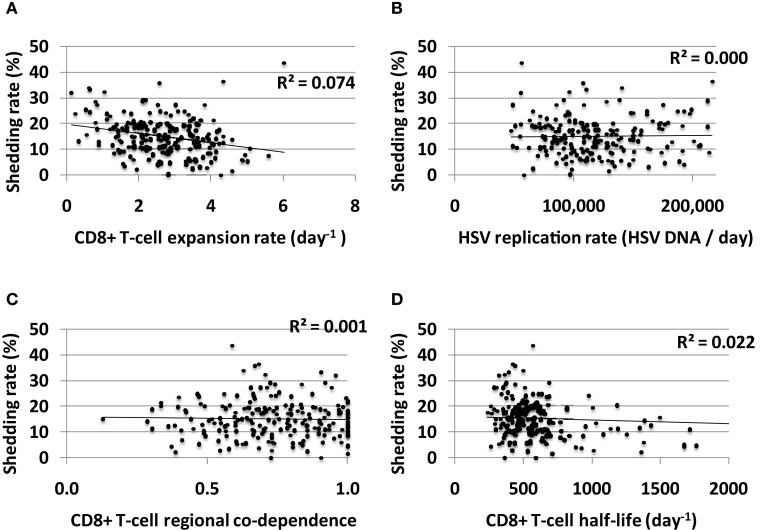
**CD8+ T-cell influenced parameters that are important mediators of short-term viral control have only a limited impact on long-term shedding rates**. **(A)** CD8+ T-cell expansion rate expansion rate predicts slightly lower shedding rate. **(B)** HSV DNA replication rate has no impact on shedding rate. **(C)** CD8+ T-cell regional co-dependence, a model parameter that dictates the range over which these cells provide protection, has no impact on shedding rate. **(D)** Longer CD8+ T-cell half-life weakly predicts lower shedding rate.

Increased CD8+ T-cell expansion rate predicted a slight decrease in shedding rate though this effect was limited (Figure 8A). HSV-2 replication rate in keratinocytes, CD8+ T-cell regional co-dependence (a measure of CD8+ T-cell diffusion between regions), and CD8+ T-cell half-life had little predictive effect on HSV-2 shedding rate in model simulations (Figures 8B–D). The timing of recognition of infected cells by CD8+ T-cells (parameter *r*) had no impact on shedding rate (not shown).

### Large effect of mucosal neuronal release rate of HSV-2 on long-term shedding rate

In model simulations, shedding episodes are initiated when viruses from neurons infect epithelial cells where replication and spread is explosive, allowing HSV DNA to reach the skin surface. As a result, increased shedding rate may theoretically be due to high frequency of episodes, prolonged mean episode duration, or both.

**Figure 9:**
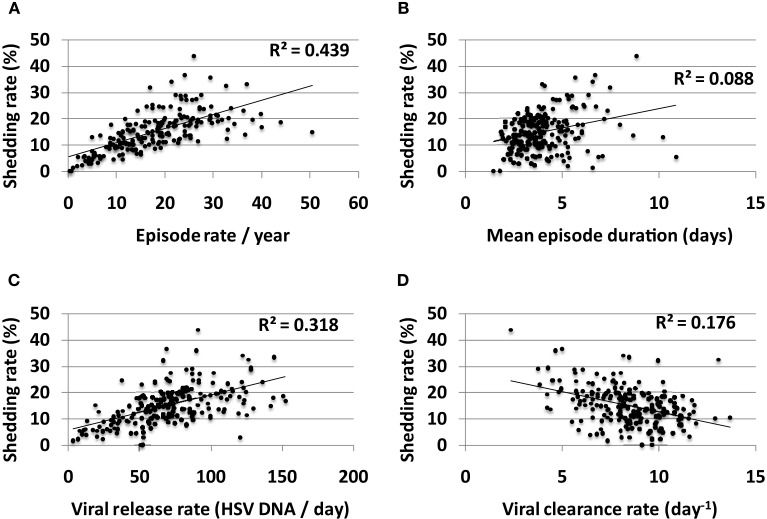
**Multiple episode initiations are the key driver of high shedding rate**. **(A)** High episode rate predicts high shedding rate to a greater extent than **(B)** high mean episode duration (which is a summary measure of mucosal control). Parameter values that allow more frequent initiation of shedding episodes in the genital tract such as **(C)** high release rate of HSV from neurons into genital skin and **(D)** low clearance rate of free HSV particles from the genital tract, are most predictive of high shedding rate.

Episode rate tightly correlated with shedding rate in simulations (Figure 9A). On the other hand, episode duration had a smaller predictive impact on shedding rate (Figure 9B). Episode initiations in the model are predicted by the stochastic term (β × *S* × *V*_neu_) with *V*_neu_ initiated at a “drip rate” of ψ and decay rate (*V*_neu_ × *c*). Therefore, three parameters might influence episode rate viral infectivity (β), neuronal drip rate (ψ), and viral clearance rate (*c*). Accordingly, both high neuronal release rate of HSV (Figure 9C) and low viral clearance rate (Figure 9D) predicted higher shedding rate. Viral infectivity also had a slight positive predictive effect on shedding rate (*R*^2^ = 0.09).

### Possible therapeutic vaccine induced effects on mucosal control of HSV-2

**Figure 10:**
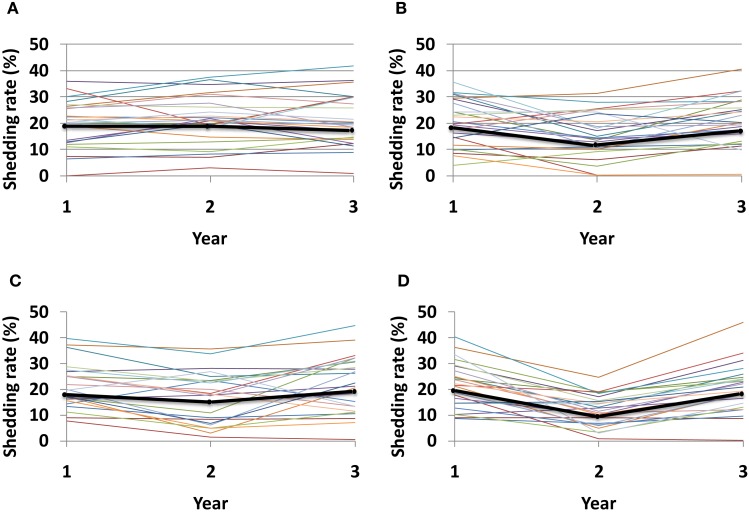
**Theoretical effect of an HSV-2 immunotherapy on yearly shedding rates**. We measured shedding rates for simulated patients with 25 parameter sets. Year 1 shedding rate represents 1-year shedding rate pre-immunotherapy. Year 2 and 3 shedding rates are averaged over the first and second year following immunotherapy, respectively. Each thin colored line represents a simulation with an individual parameter set while the thick black line represents median values for each year. Simulations assume that immunotherapy leads to **(A)** 25% increase of total CD8+ T-cells applied within each individual region, **(B)** 50% increase of total CD8+ T-cells applied within each individual region, **(C)** 25% increase of total CD8+ T-cells applied evenly across all 300-modeled regions, and **(D)** 50% increase of total CD8+ T-cells applied evenly across all 300-modeled regions. An increase in total number of recruited CD8+ T-cells **(B,D)**, as well as a more even recruitment of CD8+ T-cells **(C,D)** leads to the largest decline in shedding at year 2, though normal dynamics eventually return leading to high shedding during year 3.

Given the recently recognized importance of HSV specific mucosal CD8+ effector memory T-cells for control of HSV-2, a current therapeutic vaccine strategy is to increase the number of CD8+ T-cells in the genital mucosa. Such a strategy might induce a uniform increase in CD8+ T-cells, regardless of initial CD8+ T-cell levels within specific micro-regions. Alternatively, increased CD8+ T-cell levels may depend on initial levels within each region if the immunotherapy induces local replication of pre-existing cells. Under this set of rules, any increase in local CD8+ lymphocyte levels is relative to prior levels. Simulations demonstrate a dose response for increase in number of tissue resident CD8+ T-cells, and an added benefit for even distribution of recruited CD8+ T-cells rather than focal accumulations at prior regions with high levels (Figure 10).

## Discussion

I used a mathematical model to interrogate different components of the immune response to a spatially dispersed frequently relapsing infection and identified that increased genital tract T-cell inflammation does not necessarily reflect better control of infected cells. During the course of natural infection, numerous dense focal islands of mucosal lymphocytes may indicate recent episodes of breakthrough shedding rather than evidence of comprehensive protection against subsequent mucosal reactivation. Even when total levels of HSV-2 specific CD8+ lymphocytes in the genital tract are high, the model predicts, that regions with low density predominate allowing breakthrough replication. This result does not alter the fact that a high density of HSV specific T-cells in mucosa might be a critical outcome for an immunotherapy or a vaccine, but rather highlights the high degree of host/viral co-dependence during unperturbed, chronic infection.

The model results also provide new hypotheses regarding shedding heterogeneity among infected persons. Episode rate is a key driver of shedding rate in simulations, a trend that is also observed in empirical datasets (27). Factors, which promote more frequent episode initiation, the most important of which may be rate of viral release from ganglia, are important therapeutic targets. For instance, ganglionic CD8+ T-cells may exert control on HSV reactivation via non-cytolytic means (31, 32). Surprisingly, parameters of enhanced CD8+ T-cell functionality in mucosa that correlate with better short-term containment of HSV may not mediate enhanced long-term decreases in shedding rate. However, it is unknown if the breadth and specificity of immunity in ganglia and mucosa are mediated independently. Therefore, immune priming in both neuronal and mucosal compartments may be an important goal in development of immunotherapies (33).

Several caveats apply to these results. Mathematical models are similar to animal models of infection in that they represent simplified abstractions of complex viral host interactions in humans. As such, the model employed in this paper is a hypothesis generation tool. My model, while mathematically complex, is immunologically simple, and negates most features of the highly coordinated mucosal response including antigen presentation, CD4+ T-cell help and innate responses. Moreover, I assume the possibility of heterogeneity for all parameter values. In truth, certain parameters are likely to be far more variable between infected persons than others. However, there is a dearth of available information to define these characteristics for human infections as most immunologic measures are made in cross section rather than serially across spatially complex microenvironments. As such, there is no way at present to understand whether key parameters such as CD8+ T-cell expansion rate or viral replication rate are stable or variable in an uninfected person over time.

In addition, the predator prey structure of the model (with CD8+ T-cells as predator and infected cells as prey) is critical to its predictions regarding frequent CD8+ T-cell reconstitution in genital tract, but is still based on theory. Indeed, predator prey dynamics are not relevant for all types of immunity: the systemic, humoral arm of the immune system appears to provide a durable response over decades in the absence of antigenic re-stimulation (34, 35). However, for HSV-2 there is sufficient proof to structure the model with the predator prey assumption: CD8+ T-cells locally expand following a viral replication ulcer, and decay in tissue slowly once virus is cleared (12, 13). The continued availability of prey (virus) is documented in studies from hundreds of patients showing repeated episodic shedding over short time scales of days to weeks. The spatial component of our model, which is also based on detailed observations of gradients in viral and CD8+ T-cell quantity is tissue, has the potential to alter the predator prey dynamics. Therefore, the dependence of inflammation quantity (predator) on viral shedding (prey) is not a tautology of the model, but rather a fundamental emergent property of simulations with multiple parameter sets.

With these reservations in mind, the model’s results still serve an important purpose. Like other human viruses, HSV-2 replicates in a non-linear microenvironment with complex spatial constraints. At minimum, these model results are a warning to immunologists and vaccinologists to avoid certain traps that may arise from over interpreting studies with cross-sectional design. First, results from studies that use single, non-mechanistic measures of immunity as correlates of less severe outcome, should not be used to infer causality. Identifying true pathways of immune control in tissue requires careful studies with serial sampling, as well as mathematical approaches to account for temporal and spatial non-linearity in the system.

The limitations of human natural history studies must be acknowledged: conditions cannot be controlled in observational cohorts and readout measures of infection severity are complex. Animal models limit the complexity by focusing precisely on pre-determined exposure variables and outcome measures. Yet, infection conditions are artificial and fail to capture the viral dynamics that represent co-evolution between virus and host over millennia. Modeling is an ideal method to analyze serial, observational human data. Moreover, hypotheses raised in animal systems can be tested for their applicability to human systems using competing mathematical models.

This study highlights a new approach toward considering shedding heterogeneity in HSV-2 infection that is widely applicable to other human infections. Rather than focus on a specific molecular pathway, which correlates with severe disease in a minority of infected people, we attempt to couch heterogeneity according to definable parameters of CD8+ T-cell functionality and viral replication. The surprising result, that improvements in short-term immune control may not correlate with improved long-term outcomes, highlights the need for further studies in this discipline, as well as for further collaborations between theorists and empirical immunologists.

## Conflict of Interest Statement

The authors declare that the research was conducted in the absence of any commercial or financial relationships that could be construed as a potential conflict of interest.
